# The adjuvant value of Herba Cistanches when used in combination with statin in murine models

**DOI:** 10.1038/s41598-017-10008-7

**Published:** 2017-08-24

**Authors:** Elaine Wat, Chun Fai Ng, Chi Man Koon, Cheng Zhang, Si Gao, Brian Tomlinson, Clara Bik San Lau

**Affiliations:** 1 Institute of Chinese Medicine, The Chinese University of Hong Kong, Shatin, New Territories Hong Kong; 2 State Key Laboratory of Phytochemistry and Plant Resources in West China, The Chinese University of Hong Kong, Shatin, New Territories Hong Kong; 3 Division of Clinical Pharmacology, Department of Medicine and Therapeutics, The Chinese University of Hong Kong, Shatin, New Territories Hong Kong

## Abstract

Statins are well known to have muscle toxicity problem. Herba Cistanches (HC) is a Chinese herb traditionally used for pain in the loins and knees. Our previous *in vitro* study suggested that it could protect against statin-induced muscle toxicity. However, its *in vivo* protective effect has never been investigated. The objective of this study was to determine if the aqueous extract of HC (HCE) could prevent simvastatin-induced muscle toxicity in rats, and whether HCE could also exert beneficial effects on reducing high-fat diet-induced hypercholesterolemia and elevated liver cholesterol, thereby reducing the dose of simvastatin when used in combined therapy. From our results, HCE significantly restored simvastatin-induced reduction in muscle weights and reduced elevated plasma creatine kinase in rats. HCE also improved simvastatin-induced reduction in muscle glutathione levels, muscle mitochondrial membrane potential, and reduced simvastatin-induced muscle inflammation. Furthermore, HCE could exert reduction on liver weight, total liver lipid levels and plasma lipid levels in high-fat-fed mice. In conclusion, our study provided *in vivo* evidence that HCE has potential protective effect on simvastatin-induced toxicity in muscles, and also beneficial effects on diet-induced non-alcoholic fatty liver and hyperlipidemia when being used alone or in combination with simvastatin at a reduced dose.

## Introduction

HMG-CoA reductase inhibitors, also known as statins, are well documented to be beneficial to hypercholesterolemic patients with moderate and high cardiovascular disease risk^1^. Statins, one of the best selling prescription drug class in the world, act via inhibiting the reduction of HMG-CoA to mevalonic acid during the early stage of the mevalonate pathway to reduce endogenous cholesterol synthesis^2, 3^. Although statins are usually well tolerated, one of the most important and well known clinical adverse effects is skeletal muscle abnormalities, which can range from benign myalgia to severe myopathy^4, 5^. In a large survey on 10,409 French subjects conducted through telephone interview, 10% of the patients receiving statin treatment reported muscular symptoms, from which 30% of these symptomatic patients resulted in treatment discontinuation^6^. In fact, the National Lipid Association Statin Safety Task Force has provided recommendations for the management of muscle-related symptoms in patients undergoing statin therapy. In general, it is advised that in patients with severe symptoms such as elevated creatine kinase (CK) of more than 5 × ULN, statins should be withheld, until the CK level is normalized again^5^. Due to the occurrence of these side effects, and that no effective treatment exists for statin-induced muscle toxicity, there is thus an urge to search for new therapies for the management of the statin-induced muscle problems.

While a medley of mechanisms are proposed and could be responsible for the adverse effects of statin, mitochondrial mechanisms are believed to be implicated in the muscle toxicity adverse effects of statin^1^. Mevalonate is not only a cholesterol precursor but is also precursors for important compounds, such as selenoproteins, dolichol, and ubiquinone^7, 8^. Statin can down-regulate selenoprotein such as glutathione peroxidase (GPx), compromising the antioxidant dense and contributing to its adverse effects^4^. Statins could also lead to ubiquinone depletion, causing reduced oxygen consumption and ATP synthesis^9^. Furthermore, increasing *in vitro* and *in vivo* studies demonstrated that statin could also act directly on tissue mitochondria causing increased reactive oxygen species (ROS) and mitochondrial oxidative stress, leading to cell death and therefore contributing to liver and muscle injury^10, 11^.

Herba Cistanches, the whole dried plant of *Cistanche deserticola* Y.C. Ma (Orobanchaceae family), are parasitic plants which predominantly grow in the desert areas of north and northeast China^12^. According to the Chinese medicine theory, Herba Cistanches has the taste of sweet, warm and salty^13^. It is a Yang-invigorating Chinese tonic herb that is primarily used to treat kidney deficiency with symptoms such as impotence, infertility, premature ejaculation. It is also a Chinese herb that is traditionally prescribed to patients for pain in the loins and knees, and is commonly used in Chinese formulations for the treatment of muscle problems^12, 14^. Interestingly, this is also consistent with the modern scientific studies which demonstrated the anti-fatigue activities of a polysaccharide-rich and phenylethanoid-rich extract of Herba Cistanches in rats post-exercising by decreasing muscle damage and improving ATP storage^15^. Besides, recent work demonstrated that a methanol extract of Herba Cistanches could enhance mitochondrial ATP generation^16^. Herba Cistanches was also proven to be a strong antioxidant and free radical scavenger in various organs, reducing the oxidative stress and ROS activities in *in vivo* studies^17, 18^. In a recent study conducted by our laboratory, we had demonstrated that our Herba Cistanches aqueous extract significantly prevented simvastatin-induced toxicity in L6 skeletal muscle cells, as well as dose-dependently restored simvastatin-induced reduction in ATP production in L6 cells, suggesting the potential of Herba Cistanches aqueous extract in protecting against statin-induced muscle toxicity^19^. Nonetheless, the *in vivo* evidence is lacking.

Hence, we hypothesized that Herba Cistanches aqueous extract (HCE) could exert beneficial effects on simvastatin-induced muscle toxicity. Thus, the aim of this study was to determine whether the use of HCE could reduce simvastatin-induced muscle toxicity using an *in vivo* animal model, and in addition, whether HCE could also exert beneficial effects on reducing high-fat diet-induced hypercholesterolemia and elevated liver cholesterol, thereby reducing the dose of simvastatin used.

## Materials and Methods

### Herbal materials authentication and extraction

Raw herbal material of Herba Cistanches (*Cistanche deserticola*, sourced from the Inner Mongolia) was purchased from ZiXin, a renowned supplier in Guangzhou, China. Herba Cistanches was chemically authenticated using thin layer chromatography and ultra performance liquid chromatography in accordance to Chinese Pharmacopoeia 2010^13^ as previously described^19^, with verbascoside and echinacoside (purchased from the National Institute for the Control of Pharmaceutical and Biological Products, China) as the chemical markers. Upon chemical authentication, herbarium voucher specimen of Herba Cistanches was deposited at the museum of the Institute of Chinese Medicine at the Chinese University of Hong Kong, with voucher specimen number as 2014-3434.

Herba Cistanches aqueous extract was prepared as described previously^19^. Briefly, 1 kg raw herb was soaked for 1 hour, followed by extraction twice by heating for 1 hour under reflux at 100 °C using 10x distilled water. The aqueous extracts (HCE) were then combined and filtered, and the filtrate was concentrated under reduced pressure at 60 °C. The concentrated extract was lyophilized to dryness. The percentage of yield was 50.1% w/w. All extracted powder was vacuum packed and stored until use.

### Experimental animals

Simvastatin was purchased from SR Pharmasolutions Ltd. Male Sprague Dawley rats (180–200 g) and male C57Bl/6J mice (8-week old) were supplied by the Laboratory Animal Services Centre, the Chinese University of Hong Kong. The animal experiments were approved by the Animal Experimentation Ethics Committee of the Chinese University of Hong Kong (Ref no. 12/074/MIS). All experimental methods were carried out in accordance with the approved guidelines specified by the Animal Experimentation Ethics Committee of the Chinese University of Hong Kong. All rats (3 animals per cage) and mice (5 animals per cage) were housed in normal standard cages at a constant temperature of 21 °C with a 12-h light-dark cycle. Each standard cage contained aspen as the bedding material. All animals were allowed *ad libitum* access to diet and water.

### Study of statin-induced muscle toxicity in rats

All rats were randomly divided into 5 groups (n = 5–10). All animals were on normal-chow diet. They were also given distilled water or simvastatin daily, and with or without HCE treatment intragastrically. For animals that are required to be given both simvastatin and HCE, HCE was given 3 hours after simvastatin administration to prevent any direct herb-drug chemical interaction. The herbal extract or simvastatin was dissolved in 0.5% methyl cellulose in distilled water at a known concentration. A pre-calculated volume was intragastrically given to each animal according to the individual’s weight so that the calculated volume contained the amount of proposed dose required. Details of these groups are as follows:

Group a) Normal-chow diet + distilled water (normal control group)

Group b) Normal-chow diet + simvastatin (640 mg/kg)

Group c) Normal-chow diet + simvastatin (640 mg/kg) + low dose HCE (1.1 g/kg of animal)

Group d) Normal-chow diet + simvastatin (640 mg/kg) + high dose HCE (2.2 g/kg of animal)

Group e) Normal-chow diet + high dose HCE (2.2 g/kg of animal)

Food intake was recorded daily and body weight was measured twice weekly. Treatment was given for 4 weeks. After that, animals were anaesthetized using a mixture of ketamine (100 mg/kg) and xylazine (10 mg/kg) intraperitoneally. Blood was withdrawn by cardiac puncture and allowed to clot. Plasma was then separated by centrifugation (3,000 rpm for 10 minutes). Various types of muscles (quadriceps, gastrocnemius, soleus, tibialis anterior, and extensor digitorum longus) were excised and stored at −80 °C until analysis.

### Plasma creatine kinase measurement

Plasma creatine kinase (CK) activity was determined using Stanbio CK Liqui-UV^®^ kit (2910-430, Stanbio Laboratory, USA) according to manufacturer’s instructions.

### Muscle glutathione peroxidase measurement

Muscle glutathione peroxidase (GPx) was measured using the commercially available kit (703102, Cayman Chemical, USA) according to manufacturer’s instructions. In brief, muscle tissues were rinsed with PBS at pH 7.4 to remove any red blood cells, and homogenised in 5–10 ml cold lysis buffer, after which the homogenate was centrifuged at 10,000 g for 15 minutes at 4 °C. Supernatant was then removed and assayed for GPx activity using the commercial kit.

### Muscle mitochondrial and cytosolic fractions preparation

Mitochondrial and cytosolic fractions from muscle samples were prepared using Mitochondria Isolation Kit (MITOISO, Sigma-Aldrich Corporation, USA) according to manufacturer’s instructions. Briefly, fresh muscle samples were pre-treated with extraction buffer, cut into small pieces and homogenised in extraction buffer. Tissue homogenates were then centrifuged. Supernatant was collected, and reserved for cytosolic fraction analysis. The mitochondrial pellets were then washed and centrifuged again and used for the mitochondrial fraction analysis.

### Muscle Mitochondrial Reactive Oxygen Species (ROS) measurement

ROS was measured with the protocol modified from previous literature^20^. Briefly, ROS was measured from the mitochondrial fraction (50 µg protein/ml) using DCFDA solution (C6827, Invitrogen, USA). The mixture was incubated at 37 °C for 10 minutes in the dark, after which it was incubated with substrate solution (10 mM pyruvate, 15 mM malate) for photo-activation. Fluorescence intensity was measured every 5 minutes for 60 minutes by BioRad Fluostar Optima 413-101 BMG Fluorescent Microplate Reader at 485 nm excitation and 520 nm emission. Fluorescence intensities were calculated for measurement of ROS generation.

### Muscle mitochondrial membrane potential

The mitochondrial membrane potential of mitochondrial fraction from muscle samples was determined according to Manufacturer’s protocol (MITOISO, Sigma-Aldrich Corporation, USA). This procedure is a fixed-point assay measuring the uptake of JC-1 with formation of the J-aggregates. Briefly, the mitochondrial suspension was diluted to contain 20 µg of protein, and was incubated with the JC-1 stain for 7 minutes at room temperature. This 7-minute is a time optimized and validated by the Manufacturer using various compounds (as stated in the manufacturer’s protocol) since the observed fluorescence of the solution will rise to a plateau after 5–10 minutes, followed by drastic decrease in the JC-1 fluorescence due to equalization of the JC-1 concentrations inside and outside the mitochondrial matrix. Fluorescence intensity was measured by BioRad Fluostar Optima 413-101 BMG Fluorescent Microplate Reader at 490 nm excitation and 590 nm emission. Fluorescence intensities were calculated for measurement of potential gradient.

### Muscle histology and inflammation assessment

Immunohistochemistry of inflammatory cells within muscles were assessed by CD68 and CD163 staining as previously described^21–23^. Paraffin sections of 5 μm were cut, adhered to Superfrost™ Plus glass slides (Menzel-Gläser, Germany). The primary mouse anti-rat CD68, ED1, and CD163 antibodies, ED2, (Bio-Rad, USA) were applied to the sections at 1:100 dilution. Specific staining was detected by the EnVision™ Systems (Dako, Denmark), using the secondary antibody, EnVision-HRP polymer-conjugated anti-mouse IgG at a dilution of 1:200, and detected using DAB solution (Dako, Denmark), followed by counterstaining with haematoxylin. Sections were then mounted in dibutyl-pthalate in histolene (DPX). The presence of neutrophils in the muscles was detected by quantitative immunohistochemistry using ImageJ software.

### Muscle gene expression analysis

PCR assay which profiles genes related to oxidative stress was performed using Rat Oxidative Stress and Antioxidant Defense PCR-arrays (PARN-065Z, SABiosciences, Frederick, USA). PCR array was performed according to manufacturer’s instruction. Briefly, total RNA was isolated by selective binding to a silica gel-based membrane following the lysis and homogenisation of muscle samples in a denaturing guanidine thiocyanate buffer (RNeasy kit, Qiagen). RNA was reverse transcribed into cDNA using the RT2 First Strand Kit (SABiosciences, Frederick, USA). This cDNA was then added to the RT2 SYBR Green qPCR Master Mix (SABiosciences, Frederick, USA). Next, each sample was aliquotted on Rat Oxidative Stress and Antioxidant Defense PCR-arrays (SABiosciences, Frederick, USA). All steps were performed according to the manufacturer’s protocol. PCR-array data were analysed using an MS-Excel sheet with macros downloaded from the manufacturer’s website (http://www.sabiosciences.com/pcrarraydataanalysis.php).

### Study of the high-fat diet-induced hyperlipidemia and hepatic steatosis in mice

All mice were randomly divided into 5 groups (n = 8–10) and received the following: Group a) Normal-chow diet (Table 1); Groups b to e) High-fat diet (contains 21% fat and 0.15% cholesterol) (Table 2). Diets were given for 8 weeks for animals to induce obesity. After 8 weeks, all high-fat fed groups were given distilled water, or simvastatin daily, and or HCE treatment intragastrically, 3 hours after simvastatin administration. The herbal extract or simvastatin was dissolved in 0.5% methyl cellulose (Sigma-Aldrich Corporation, USA) in distilled water at a known concentration. A pre-calculated volume was intragastrically given to each animal according to the individual’s weight so that the calculated volume contained the amount of proposed dose required. Details of these groups are as follows:

**Table 1:** Nutritional information for SF04-057 normal diet as used in the mice study.

Nutritional Parameters for normal Diet SF04-057	
Protein	19.0%
Total Fat	6.0%
Total Digestible Carbohydrate as defined by FSANZ Standard 1.2.8	59.9%
Crude Fibre	4.7%
AD Fibre	4.7%
Digestible Energy	16.1 MJ / Kg
% Total calculated digestible energy from lipids	14.0%
% Total calculated digestible energy from protein	21.0%

**Table 2:** Nutritional information for SF00-219 high-fat diet as used in the mice study.

Nutritional Parameters for high-fat Diet SF00-219	
Protein	19.0%
Total Fat	21.0%
Crude Fibre	4.7%
AD Fibre	4.7%
Digestible Energy	19.4 MJ / Kg
% Total calculated digestible energy from lipids	40.0%
% Total calculated digestible energy from protein	17.0%

Group a) Normal-chow diet + distilled water

Group b) High-fat diet + distilled water

Group c) High-fat diet + simvastatin (50 mg/kg)

Group d) High-fat diet + HCE (4.4 g/kg)

Group e) High-fat diet + simvastatin (25 mg/kg) + HCE (4.4 g/kg)

Food intake was recorded daily and body weight was measured twice weekly. At the end of the 16-weeks study, all mice were sacrificed after a 16-hour overnight fasting. Animals were anaesthetized by a mixture of ketamine (100 mg/kg) and xylazine (10 mg/kg) intraperitoneally. Blood was withdrawn by cardiac puncture and allowed to clot. Plasma was separated by centrifugation (3,000 rpm for 10 minutes). Livers were excised and stored at −80 °C until analysis.

### Plasma and liver lipid measurement

Plasma triglyceride, cholesterol (11488872216 and 11489232216, Roche diagnostics, Switzerland), high-density and low-density lipoprotein cholesterol (L-Type HDL-C and L-Type LDL-C, Wako Pure Chemicals Industries, Japan) concentrations were measured by enzymatic methods using the commercially available kit according to manufacturer’s instructions. Plasma creatine kinase (CK) activity was determined using Stanbio CK Liqui-UV^®^ kit (2910-430, Stanbio Laboratory, USA). Total liver lipids were determined gravimetrically after extraction by the method of Bligh and Dyer^24^. Individual hepatic lipids were quantified enzymatically (as described above) after solubilisation in isopropanol (Sigma-Aldrich, USA)^25^.

### Liver histopathology and inflammation assessment

#### Haematoxylin and eosin staining (H&E staining)

Thick sections (5 μm) of paraffin embedded tissue were cut with a microtome and placed on SuperFrost Plus^®^ microscope slides (Thermo Scientific, USA). Sections were subjected to H&E staining according to the method of Harris *et al*.^26^, and mounted with dibutyl-pthalate in histolene (DPX) (Sigma-Aldrich, USA)^25^.

#### Mac-3 staining and quantification

Thick sections (5 μm) of paraffin embedded tissue were cut using a microtome and placed on SuperFrost Plus^®^ microscope slides (Thermo Scientific, USA). Antigen retrieval was performed, followed by blocking with Peroxidase Block (Dako Cytomation, Denmark). Primary purified anti-mouse Mac-3 antibody (BD Pharmingen, USA) was applied to the sections at 1:50 dilution. Specific staining was detected using the EnVision™ Systems (Dako, Denmark). Slides were incubated with the secondary antibody, EnVision-HRP polymer-conjugated anti-rabbit IgG at a dilution of 1:200. Mac-3 was detected using DAB solution (Dako, Denmark) and counterstained with haematoxylin. Sections were then mounted in DPX. The presence of macrophages in the liver was detected by quantitative immunohistochemistry using ImageJ software.

#### Statistical Analysis

The differences among treatment and control groups were tested with one-way analysis of variance (ANOVA), followed by the post-hoc Bonferroni’s multiple comparison test. All statistical analyses were performed using GraphPad Prism Version 6.0 (GraphPad, USA). A probability of *p* < 0.05 would be considered as statistically significant.

## Results

### Effect of HCE on simvastatin-induced muscle toxicity in rat

#### Muscle weight and plasma creatine kinase measurement

**Figure 1:**
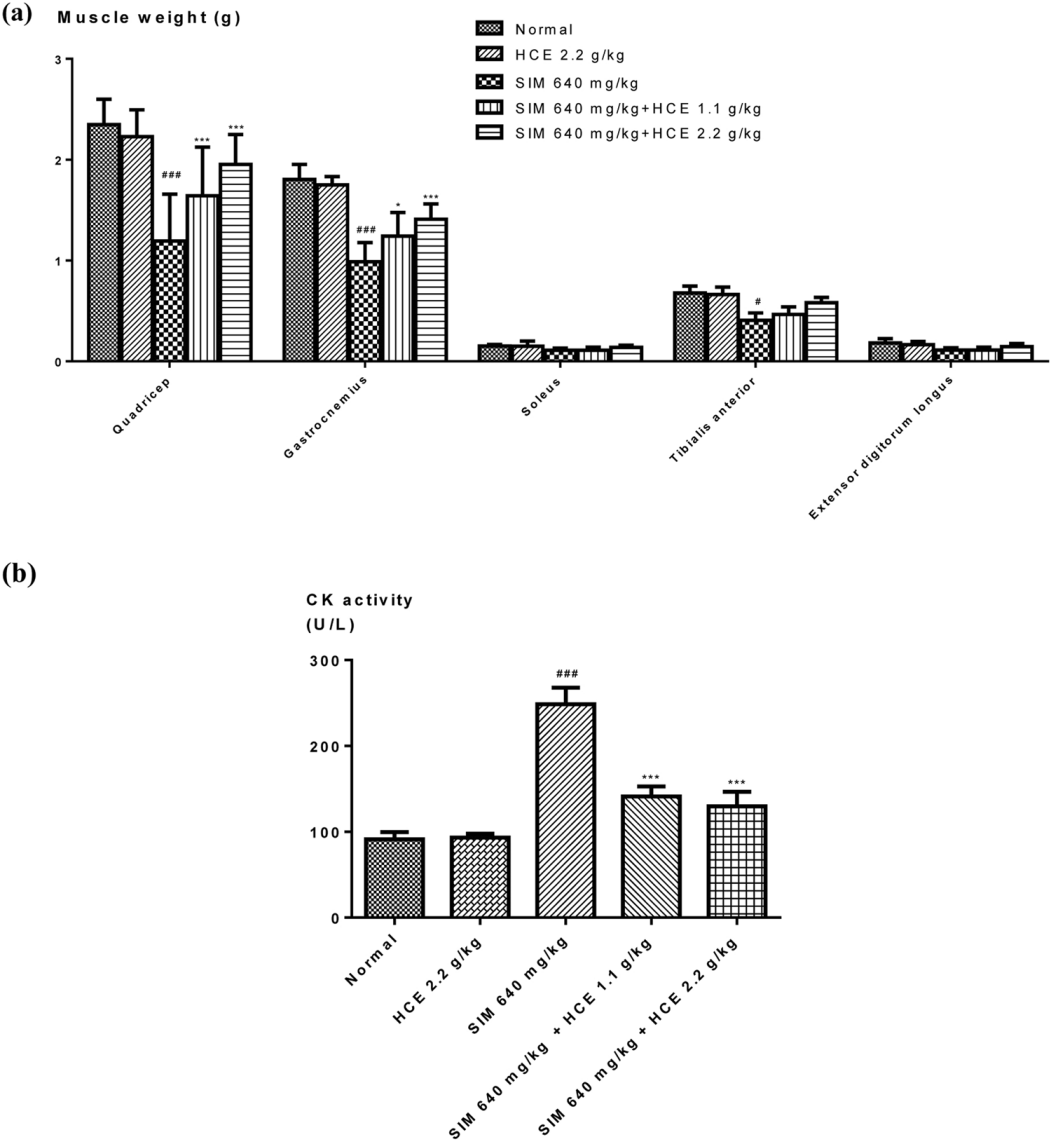
Effects of HCE on simvastatin-induced (**a**) muscle weight loss; and (**b**) creatine kinase activity in rats. Values represent means ± SEM (n = 5–10). Significant difference between simvastatin treatment alone and normal control group using Student’s *t*-test: ^#^ *p* < 0.05, ^###^ *p* < 0.001. Significant difference among simvastatin treatment alone group and simvastatin co-treatment groups with HCE using one-way ANOVA: *p < 0.05, **p < 0.01, ***p < 0.001. No significant difference was observed among the normal control group and HCE (2.2 g/kg) alone treatment group.

Five different types of muscles (quadriceps, gastrocnemius, soleus, tibialis anterior, and extensor digitorum longus muscles) were isolated from all treated rats. The effects of different treatments on muscle weight are shown in Fig. 1a. Simvastatin (640 mg/kg) induced significant weight reduction on quadriceps, gastrocnemius and tibialis anterior muscles in rats, while these reductions were significantly restored by HCE on quadriceps and gastrocnemius muscles in a dose-dependent manner. Although HCE exerted a trend to restore the weights of tibialis anterior muscle in simvastatin-treated rats, none of these treatment groups had reached statistical significance. No significant effect of HCE alone treatment on muscle weight was observed when compared to control rats (Fig. 1a).

Figure 1b showed the effects of the different treatments on plasma creatine kinase (CK) levels in rats. Simvastatin induced significant increase on creatine kinase activity in rats, which was significantly reduced by HCE co-treatments at both doses. There was also no significant effect of HCE alone treatment on CK levels as compared to the control group.

Since gastrocnemius appeared to be sensitive to simvastatin treatment and it is the largest muscle isolated by mass, we therefore chose this muscle for further analysis including muscle glutathione peroxidase measurement (GPx), mitochondrial MMP and ROS measurement, and muscle histology and inflammation assessment.

#### Muscle glutathione peroxidase (GPx) measurement

**Figure 2:**
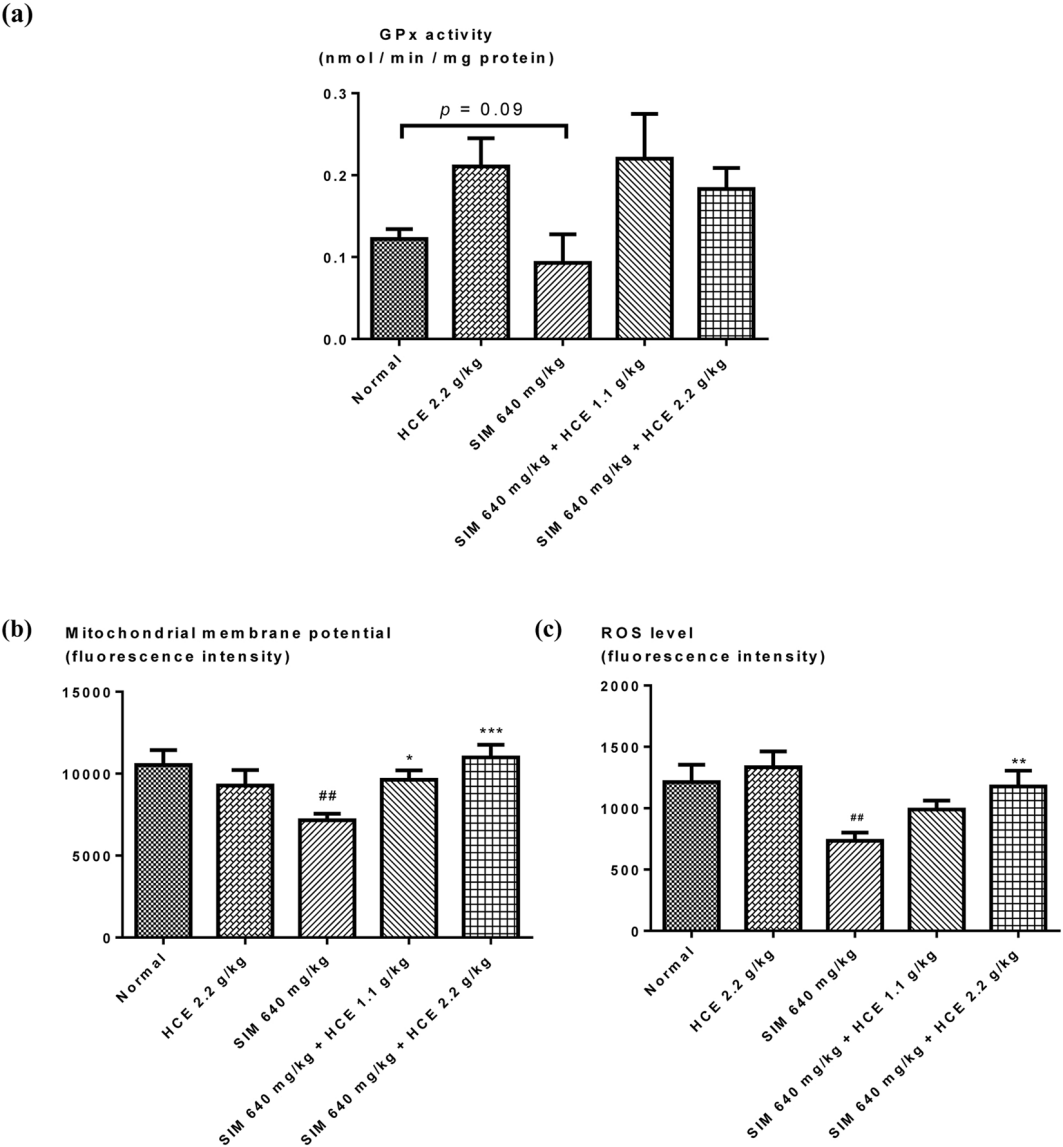
Effects of HCE on (**a**) muscle glutathione peroxidase levels (GPx); (**b**) muscle mitochondrial membrane potential (MMP); and (**c**) muscle mitochondrial reactive oxygen species (ROS) levels after simvastatin treatment. Values represent means ± SEM (n = 5–10). Significant difference between simvastatin treatment alone and normal control group using Student’s t-test: ^#^ *p* < 0.05, ^##^ *p* < 0.01. Significant difference among simvastatin treatment alone group and simvastatin co-treatment with HCE groups using one-way ANOVA: *p < 0.05, **p < 0.01, ***p < 0.001. No significant difference was observed among the normal control group and HCE (2.2 g/kg) alone treatment group.

Muscle glutathione peroxidase levels were measured and shown in Fig. 2a. Simvastatin administration induced a trend for a reduction in muscle glutathione peroxidase (GPx) measurement in rats. However, HCE exerted a trend to ameliorate the simvastatin-induced reduction in muscle GPx at both doses of 1.1 g/kg and 2.2 g/kg. There was also no significant effect of HCE treatment alone on muscle GPx levels as compared to normal control rats.

#### Mitochondrial Membrane Potential (MMP) and ROS measurement

Simvastatin (640 mg/kg) treatment induced a significant reduction in muscle MMP (Fig. 2b). This reduction was dose-dependently improved in rats receiving the co-treatment of HCE and simvastatin (Fig. 2b). On the other hand, interestingly, the simvastatin alone treatment group had significantly reduced muscle mitochondrial ROS levels compared to the normal control group (Fig. 2c). However, HCE treatment given to simvastatin-treated rats exerted a dose-dependent trend to restore muscle mitochondrial ROS levels to the normal level. There was also no significant effect of HCE treatment alone in rats compared to the normal control group for both mitochondrial MMP and ROS measurement.

#### Muscle histology and inflammation assessment

**Figure 3:**
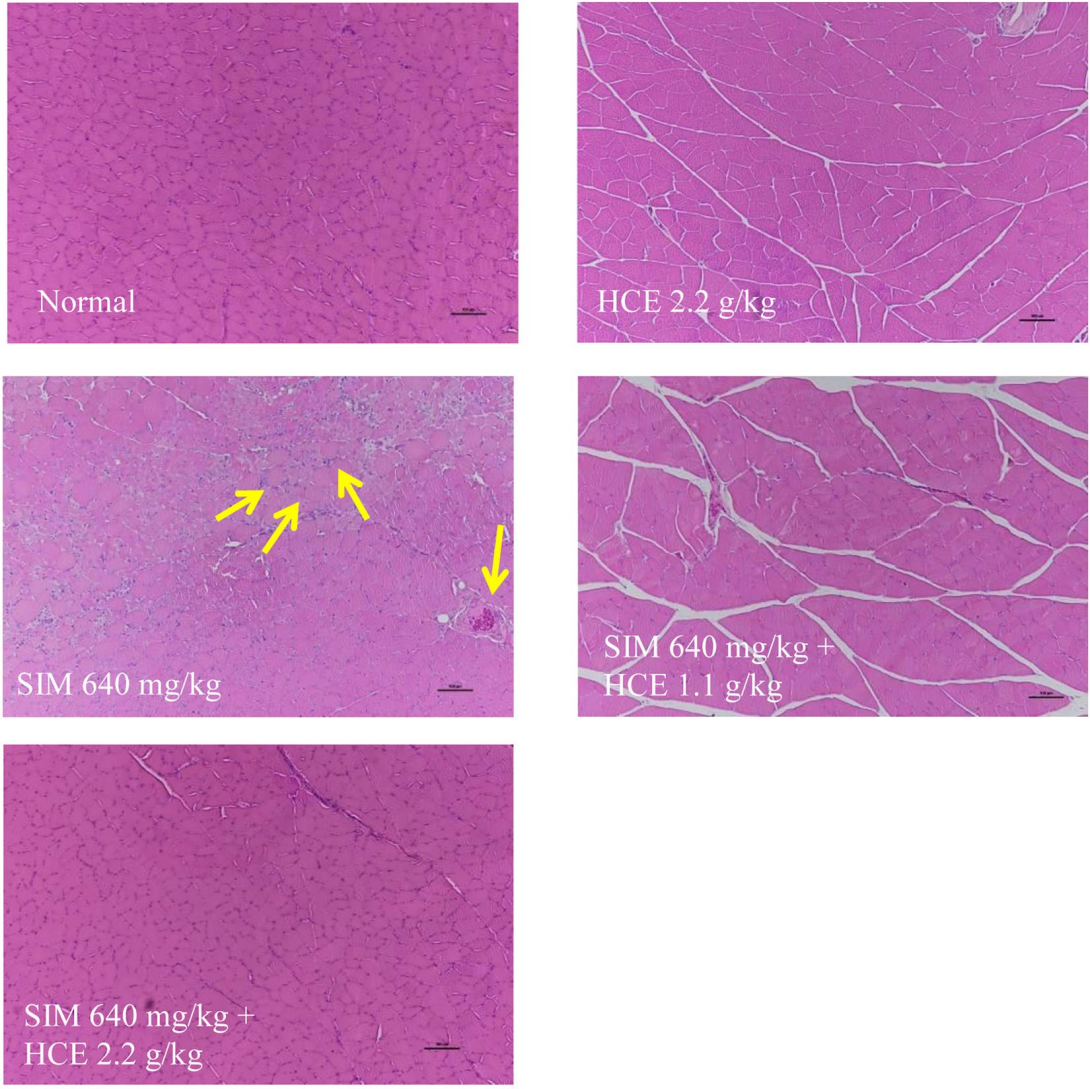
Representative gastrocnemius muscle sections (x100 magnification) from rats given different treatments and stained with haematoxylin and eosin. The yellow arrows represent infiltration of inflammatory markers. The scale bar represents 100 μm.

**Figure 4:**
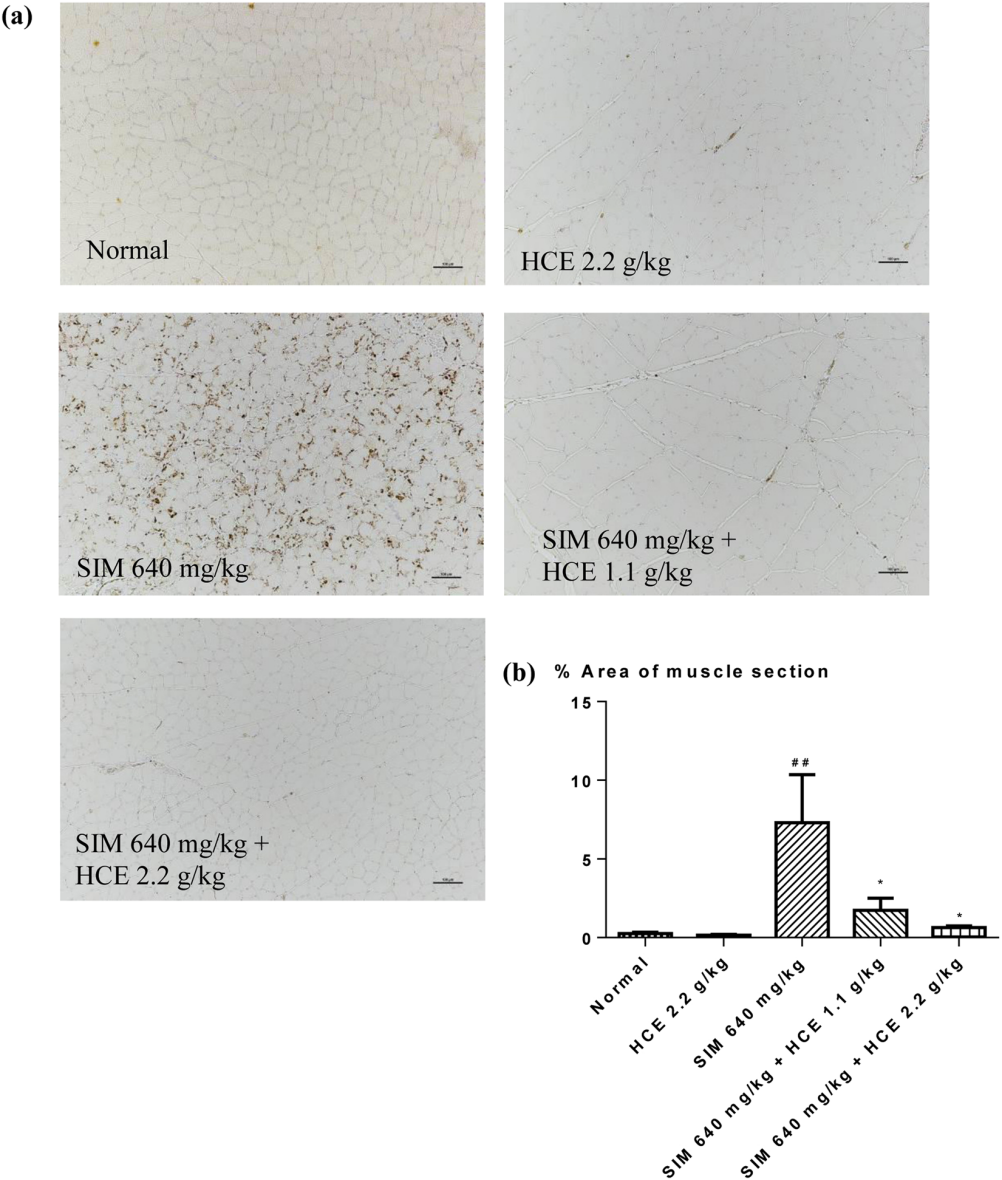
Representative gastrocnemius muscle sections (x100 magnification) from rats given different treatments and stained with (**a**) CD68; and (**b**) percentage area of gastrocnemius muscle section infiltrated by CD68. Values represent means ± SEM (n = 5–10). Significant difference between simvastatin treatment alone and normal control group using Student’s *t*-test: ^##^ *p* < 0.01. Significant difference among simvastatin alone treatment group and simvastatin co-treatment with HCE groups using one-way ANOVA: *p < 0.05. No significant difference was observed among the normal control group and HCE (2.2 g/kg) alone treatment group. The scale bar represents 100 μm.

**Figure 5:**
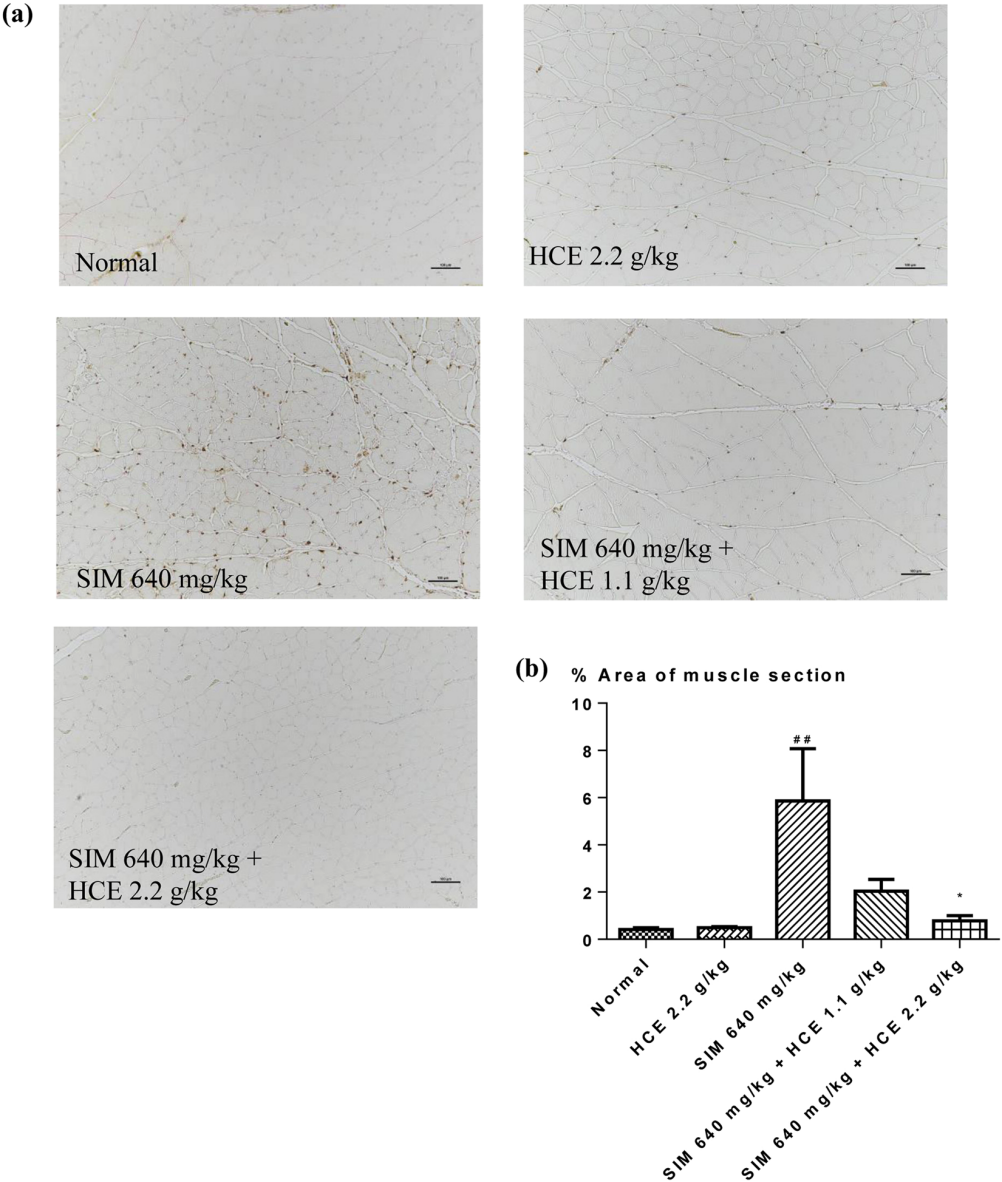
Representative gastrocnemius muscle sections (x100 magnification) from rats given different treatments and stained with (**a**) CD163; and (**b**) percentage area of gastrocnemius muscle section infiltrated by CD163. Values represent means ± SEM (n = 5–10). Significant difference between simvastatin treatment alone and normal control group using Student’s *t*-test: ^##^ *p* < 0.01. Significant difference among simvastatin alone treatment group and simvastatin co-treatment with HCE groups using one-way ANOVA: *p < 0.05. No significant difference was observed among the normal control group and HCE (2.2 g/kg) alone treatment group. The scale bar represents 100 μm.

Gastrocnemius muscles from rats given different treatments were further analyzed histologically and representative stained sections are shown in Fig. 3a. Control group animals demonstrated the histological sections of normal muscles. In contrast, H&E sections from animals treated with simvastatin revealed infiltration of inflammatory markers (yellow arrows), which was significantly reduced in those muscle sections of rats given the combination of simvastatin and HCE. Immunohistochemical analyses were also performed for the presence of macrophages CD68 and CD163 in Figs 4a and 5a, respectively, with quantifications of the respective macrophages shown in Figs 4b and 5b. Immunohistochemical analysis of muscle sections revealed the presence of macrophage (CD68) in simvastatin-treated rats (brown colouration), which was significantly reduced in rats given the HCE co-treatment at both doses (Fig. 4b). There was also no significant inflammation observed in HCE alone treated animals. Similarly, immunohistochemical analysis of muscle sections revealed the presence of CD163-positive macrophages in simvastatin-treated rats (brown colouration), which was significantly reduced in rats given the HCE co-treatment at 2.2 g/kg (Fig. 5b). There was also no significant inflammation observed in HCE alone treated animals.

#### Muscle gene expression analysis

**Figure 6:**
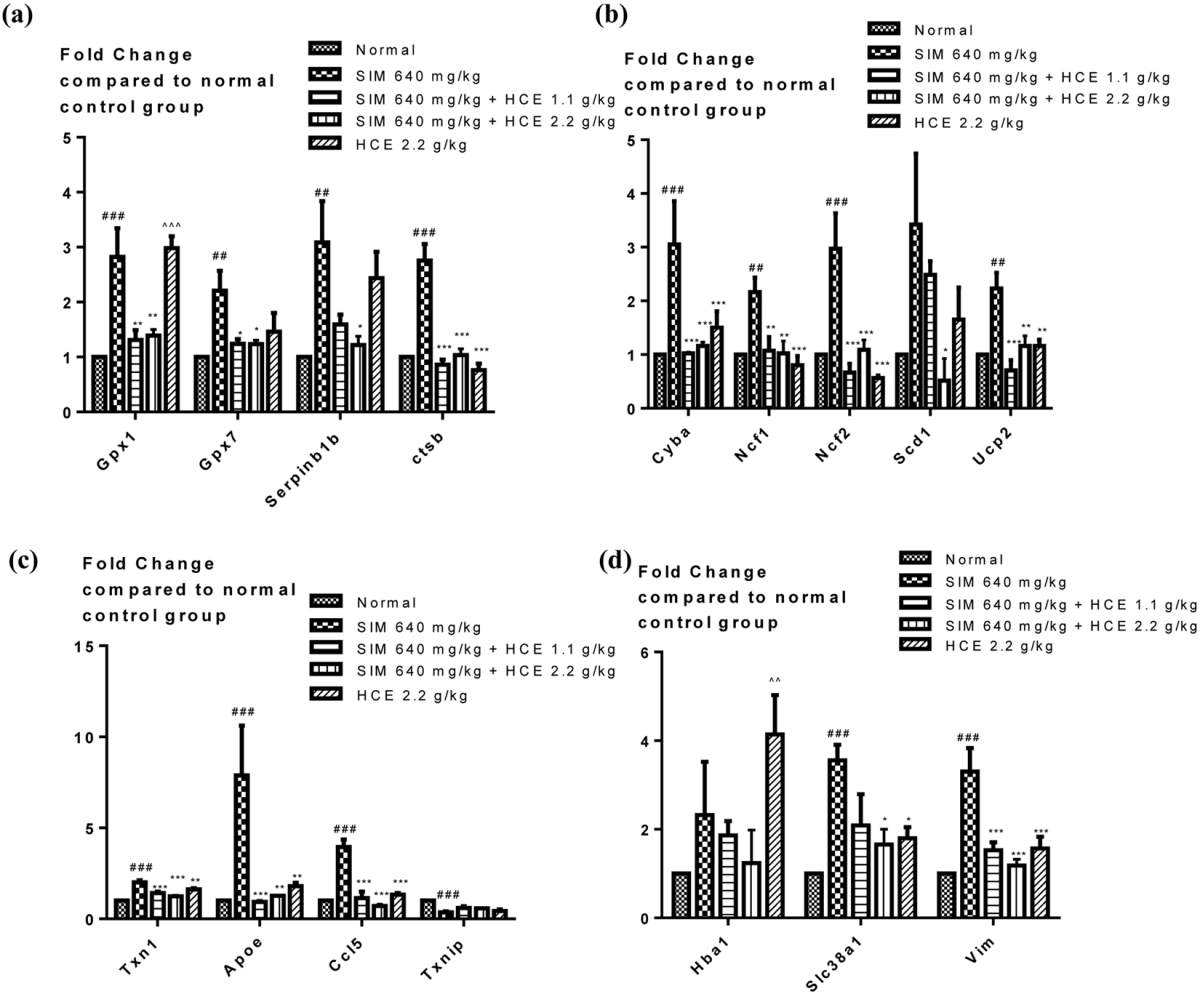
Effect of simvastatin treatment with or without HCE co-treatment on the expression levels of genes regulating (**a**) antioxidants; (**b**) superoxide metabolism; (**c**) oxidative stress response; and (**d**) oxygen transporter of rats within the muscles. Values represent means ± SEM (n = 5–10). Significant difference between simvastatin treatment alone and normal control group using Student’s *t*-test: ^##^ *p* < 0.01, ^###^ *p* < 0.001. Significant difference among simvastatin alone treatment group and simvastatin co-treatment with HCE groups using one-way ANOVA: *p < 0.05, **p < 0.01, ***p < 0.001. Significant difference among the normal control group and HCE treatment group alone using student t-test: ^^^^ *p* < 0.01, ^^^^^ *p* < 0.001.

To determine the effects of simvastatin and HCE on genes related to oxidative stress within the muscle, PCR assay which profiles genes related to oxidative stress was performed using Rat Oxidative Stress and Antioxidant Defense PCR-arrays. Genes that were significantly affected by simvastatin treatment were analysed and presented in Fig. 6. For the expression profile of genes regulating antioxidants, simvastatin (640 mg/kg) treated rats increased expression of genes regulating antioxidants. These include glutathione peroxidases (GPx) *GPx1* and *GPx7*, and other peroxidases including *Serpinb1b* and *ctsb* (Fig. 6a). HCE at both doses (1.1 or 2.2 g/kg) reduced these simvastatin-induced increase in GPxs and other peroxidases to normal levels. On the other hand, HCE treatment alone significantly increased the expression levels of *GPx1*. Regarding expression of genes regulating ROS metabolism, simvastatin significantly induced expression of other superoxide metabolism genes, including *Cyba*, *Ncf1*, *Ncf2*, *Scd1* and *Ucp2*. Similarly, HCE at both doses significantly reduced these increases (Fig. 6b). Furthermore, simvastatin induced expression of oxidative stress responsive genes including *Apoe*, *Ccl5*, and *Txn1*, while these expressions were significantly reduced in HCE co-treated animals at both doses (Fig. 6c). On the other hand, simvastatin caused a significant reduction in the gene expression of *Txnip*. There was no significant effect of HCE on the simvastatin-induced reduction in *Txnip* gene expression (Fig. 6c). Simvastatin also exerted a significant increase in expression of genes regulating oxygen transporters including *Slc38a1* and *Vim*. HCE co-treatment reduced the expression of these genes at both doses (Fig. 6d).

#### Effects of the combination use of Simvastatin and HCE on food intake and body weight in mice

**Figure 7:**
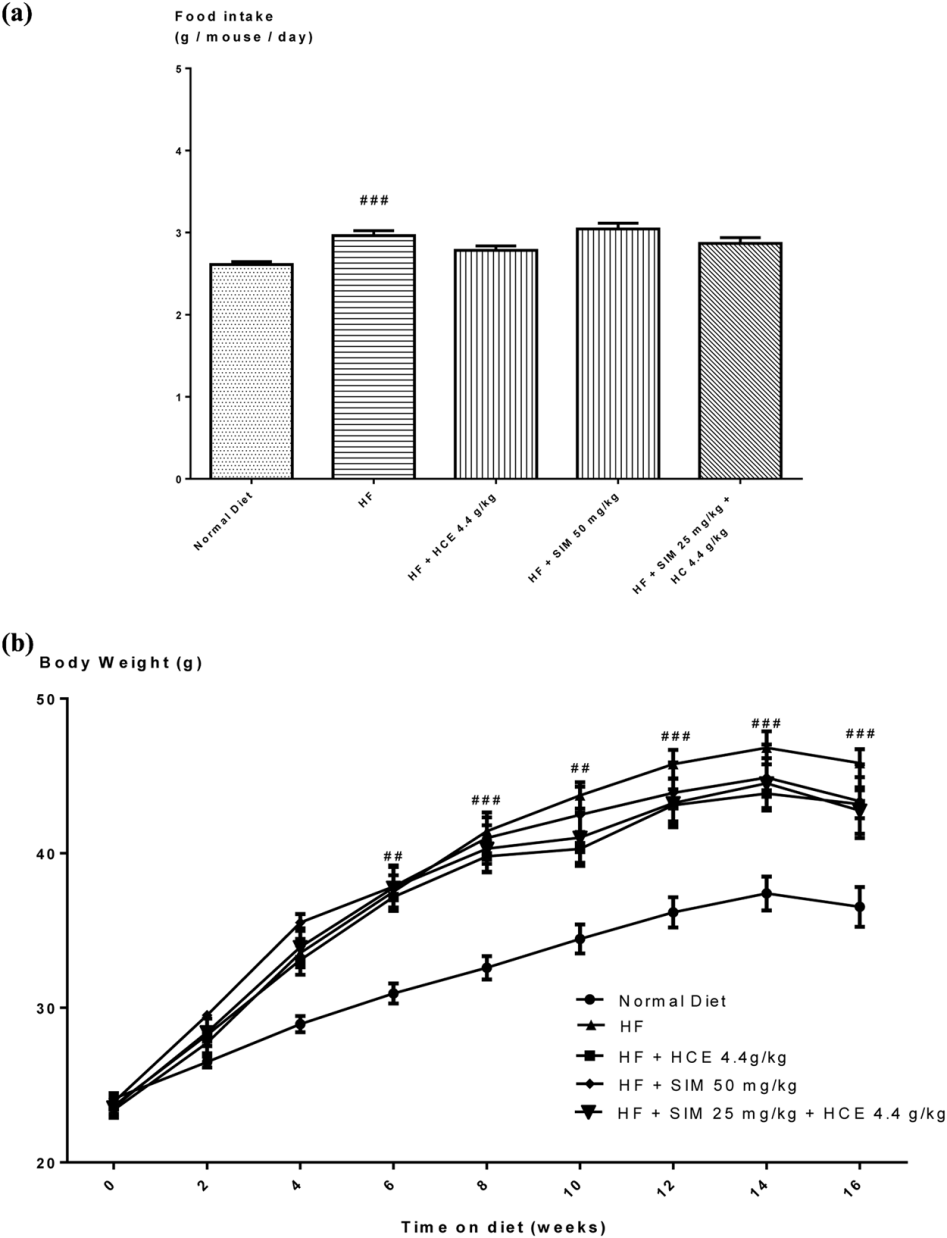
Effect of simvastatin treatment with or without HCE co-treatment in mice fed with a normal chow or high-fat diet on (**a**) daily food intake; and (**b**) weekly body weight. Values represent means ± SEM (n = 8–10). Significant difference between HF-fed and normal chow-fed group using Student’s *t*-test: ^##^ *p* < 0.01, ^###^ *p* < 0.001. No significant difference was observed among HF-fed group vs HF + SIM 50 mg/kg, or HF + HCE 4.4 g/kg, or HF + SIM 25 mg/kg + HCE 4.4 g/kg groups. No significant difference was observed among HF + SIM 25 mg/kg + HCE 4.4 g/kg, HF + HCE 4.4 g/kg, and HF + SIM 50 mg/kg groups.

Figure 7a showed the daily food intake of mice for all treatment groups. High-fat diet induced a significant increase in the daily food intake of animals compared to chow-fed animals (*p* < 0.001). There was however no significant effect of the herb or drug treatments among all high-fat-fed groups (Fig. 7a).

#### Weekly body weight measurement

After 16 weeks of high-fat feeding, HF-fed animals significantly gained more weight than chow-fed animals (Fig. 7b). However, there was no significant effect between HF-fed mice and all HF-fed receiving treatment groups. There was also no significant difference between HF-fed mice receiving the co-treatment of simvastatin and HCE vs HF-fed mice receiving HCE or simvastatin treatment alone (Fig. 7b).

### Effects of the combination use of Simvastatin and HCE on hyperlipidemia and hepatic steatosis in mice

#### Plasma lipid and creatine kinase activity measurement

**Figure 8:**
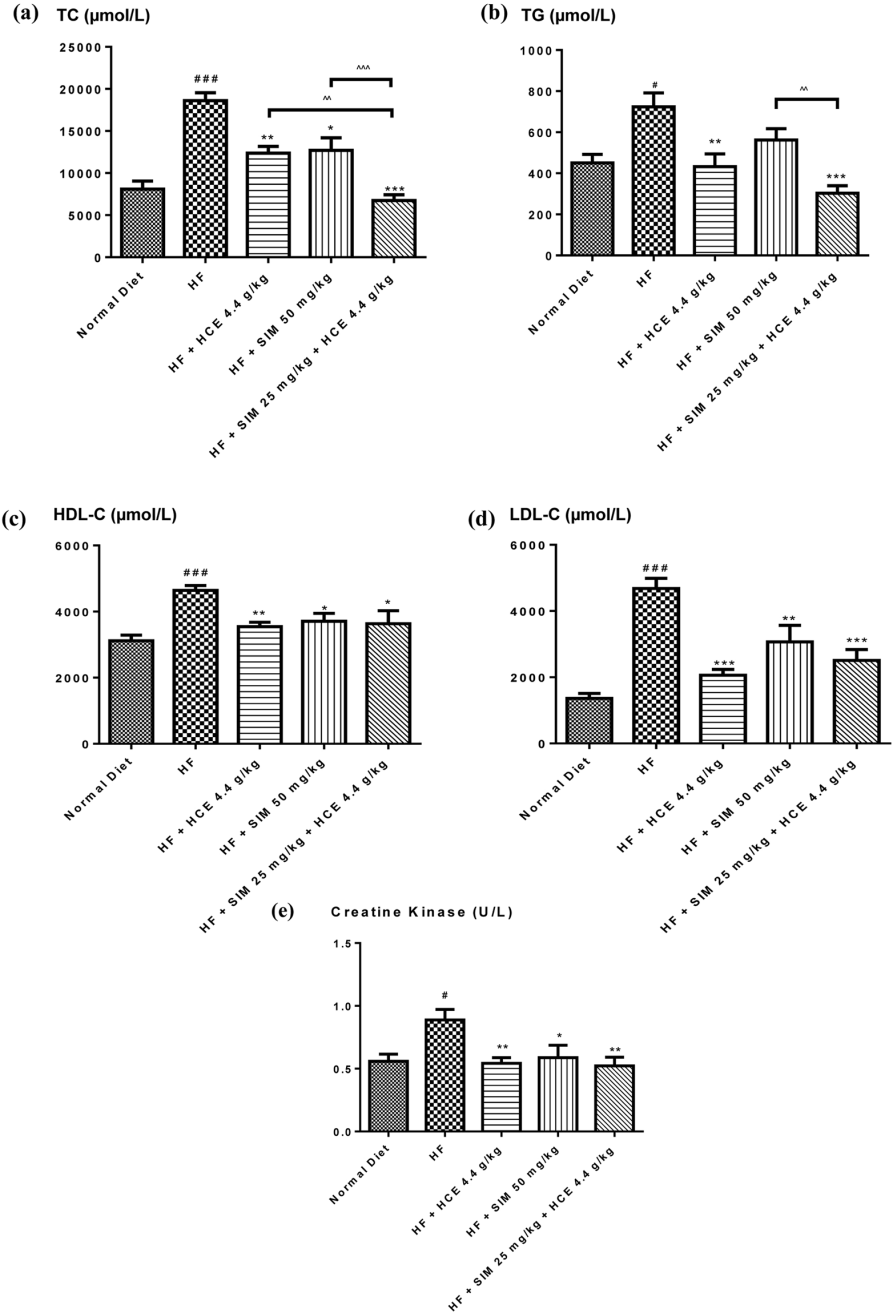
Effect of simvastatin treatment with or without HCE co-treatment in mice fed with a normal chow or high-fat diet on (**a**) plasma total cholesterol (TC); (**b**) plasma triglyceride (TG); (**c**) plasma high-density-lipoprotein cholesterol (HDL-C); (**d**) plasma low-density-lipoprotein cholesterol (LDL-C); and (**e**) plasma creatine kinase levels. Values represent means ± SEM (n = 8–10). Significant difference between HF-fed and normal chow-fed group using Student’s *t*-test: ^#^ *p* < 0.05, ^###^ *p* < 0.001. Significant difference among HF-fed group vs HF + SIM 50 mg/kg, or HF + HCE 4.4 g/kg, or HF + SIM 25 mg/kg + HCE 4.4 g/kg groups using one-way ANOVA: *p < 0.05, **p < 0.01, ***p < 0.001. Significant difference among HF + SIM 25 mg/kg + HCE 4.4 g/kg, HF + HCE 4.4 g/kg, and HF + SIM 50 mg/kg groups using one-way ANOVA: ^^^^ *p* < 0.01, ^^^^^ *p* < 0.001.

Plasma lipid levels of all mice are shown in Fig. 8. Compared to normal chow-fed mice, HF-fed mice had significantly elevated levels of plasma total cholesterol (TC), which were significantly reduced in all treatment groups (Fig. 8a). HF-fed mice treated with simvastatin and HCE had significantly lower plasma TC compared to HF animals given simvastatin alone or HF animals given HCE alone (*p < *0.001 and *p < *0.01, respectively) (Fig. 8a). No significant difference was observed between HF-fed mice receiving HCE alone vs simvastatin alone treatment group, suggesting HCE has comparable effects as simvastatin. Besides, HF-fed mice also had significantly higher plasma triglyceride (TG) levels compared to mice given normal chow diet (*p < *0.05) (Figure 8b). There was a trend for a reduction in simvastatin treatment alone group on plasma TG in HF-fed animals, though this did not reach statistical significance. However, HCE treatment, with or without simvastatin co-treatment significantly reduced plasma TG compared to control HF-fed animals (*p < *0.001 and *p < *0.01, respectively). When comparing between simvastatin treatment alone group with simvastatin co-treatment with HCE group, HCE co-treatment with simvastatin had significantly lower plasma TG compared to HF-fed mice given simvastatin treatment alone (*p* < 0.01) (Fig. 8b). No significant difference was observed between HF-fed mice receiving HCE alone vs the simvastatin treatment group, though HCE had a slightly better reduction in TG levels. In addition, high-fat-diet induced significant increase in plasma high-density lipoprotein (HDL) cholesterol and plasma low-density lipoprotein (LDL) cholesterol in mice compared to normal chow-fed animals (Fig. 8c and d). Simvastatin treatment alone significantly reduced both lipids in high-fat-fed mice. HCE treatment, with or without simvastatin co-treatment also significantly reduced both types of plasma lipids compared to mice given HF diet alone. There was no significant difference on both lipids among all treatment groups. Figure 8e showed the effect of different treatments on plasma creatine kinase (CK) levels. Interestingly, HF diet induced significant increase in plasma CK levels in mice compared to normal-chow-fed mice (Fig. 8e). HCE treatment, with or without the co-treatment of simvastatin significantly reduced plasma CK levels compared to mice given HF diet alone (*p < *0.01 and *p < *0.01, respectively). Simvastatin treatment alone also significantly reduced high-fat diet-induced increase in plasma CK levels. There was also no significant difference on plasma CK levels among all active treatment groups.

#### Liver lipid measurement

**Figure 9:**
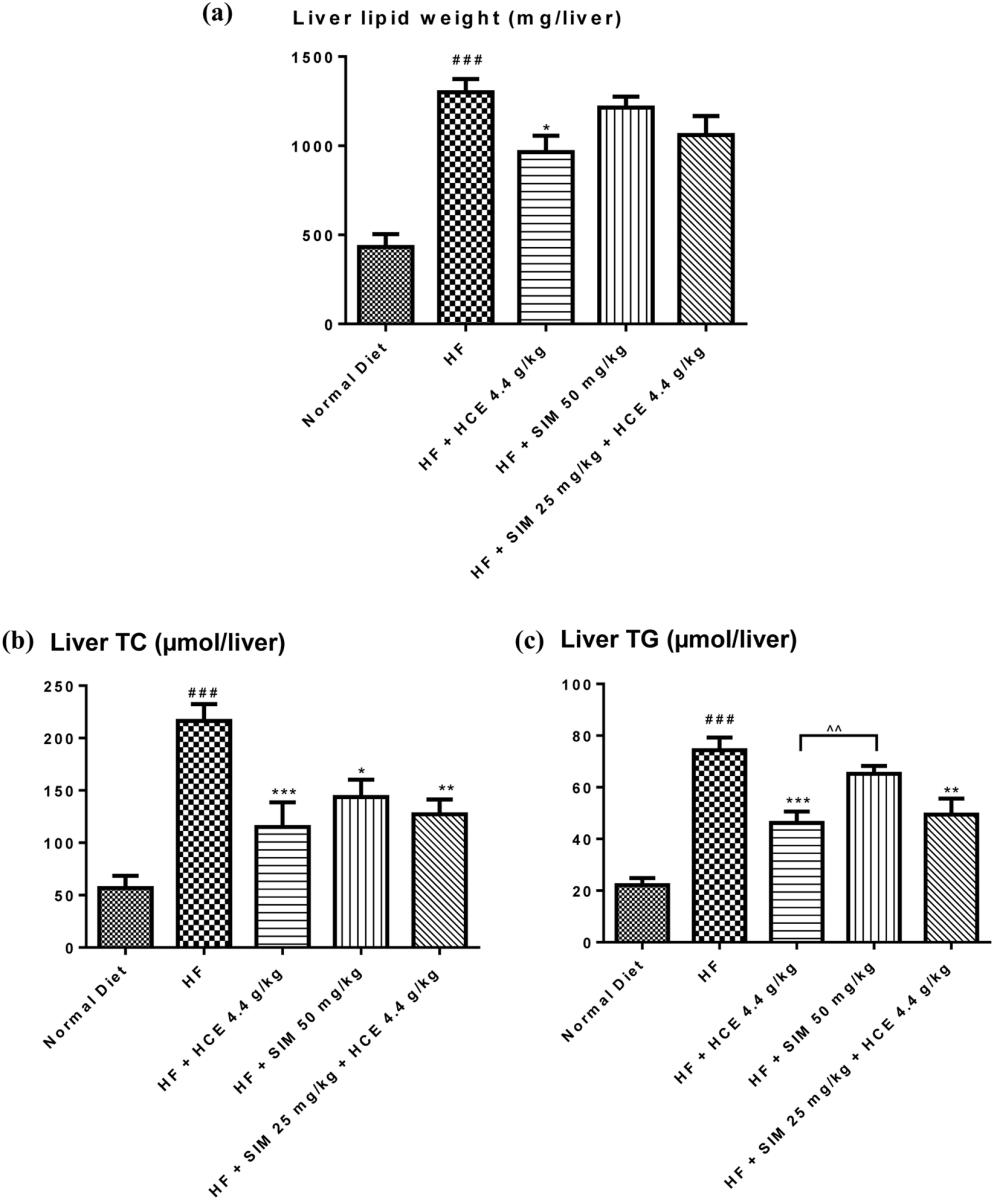
Effect of simvastatin treatment with or without HCE co-treatment in mice fed with a normal chow or high-fat diet on (**a**) total liver lipid weight; (**b**) liver total cholesterol; and (**c**) liver triglyceride levels. Values represent means ± SEM (n = 8–10). Significant difference between HF-fed and normal chow-fed group using Student’s *t*-test: ^###^ *p* < 0.001. Significant difference among HF-fed group vs HF + SIM 50 mg/kg, or HF + HCE 4.4 g/kg, or HF + SIM 25 mg/kg + HCE 4.4 g/kg groups using one-way ANOVA: *p < 0.05, **p < 0.01, ***p < 0.001. Significant difference among HF + SIM 25 mg/kg + HCE 4.4 g/kg, HF + HCE 4.4 g/kg, and HF + SIM 50 mg/kg groups using one-way ANOVA: ^^^^ *p* < 0.01.

The effects of the different treatment groups on HF-induced hepatomegaly (enlarged liver) was associated with a significant reduction in total hepatic lipid content, expressed as milligram of lipid per liver (Fig. 9a). Total liver lipid content was significantly higher in HF-fed mice compared to normal chow-fed mice (3.0-fold higher, *p* < 0.001). Simvastatin exerted no significant effect on the high-fat diet-induced increase in total liver lipid. Interestingly, HCE significantly reduced such diet-induced increase in liver lipid (*p* < 0.05). On the other hand, simvastatin co-treatment with HCE exerted a trend to reduce the liver lipid content though did not reach statistical significance (*p* = 0.15). However, when comparing the liver lipid content among all treatment groups, no significant difference was observed among all groups.

As shown in Fig. 9b and c, measurement of liver total cholesterol (TC) and liver triglyceride (TG) revealed that HF-fed mice had elevated levels of both lipids compared to normal chow-fed mice (i.e. 3.8-fold increase in TC, *p* < 0.001; and 3.4-fold increase in TG, *p* < 0.001). Simvastatin treatment significantly reduced liver total cholesterol levels (*p* < 0.05) but not triglyceride levels. On the other hand, HCE treatment, with or without simvastatin co-treatment, significantly reduced both liver total cholesterol and triglyceride levels in high-fat-fed mice. When comparing the effects among all groups, we observed no significant difference on liver total cholesterol, but there was significant reduction in liver TG for the HCE treatment group as compared to the simvastatin treatment group (Fig. 9b and c).

#### Liver inflammation assessment

**Figure 10:**
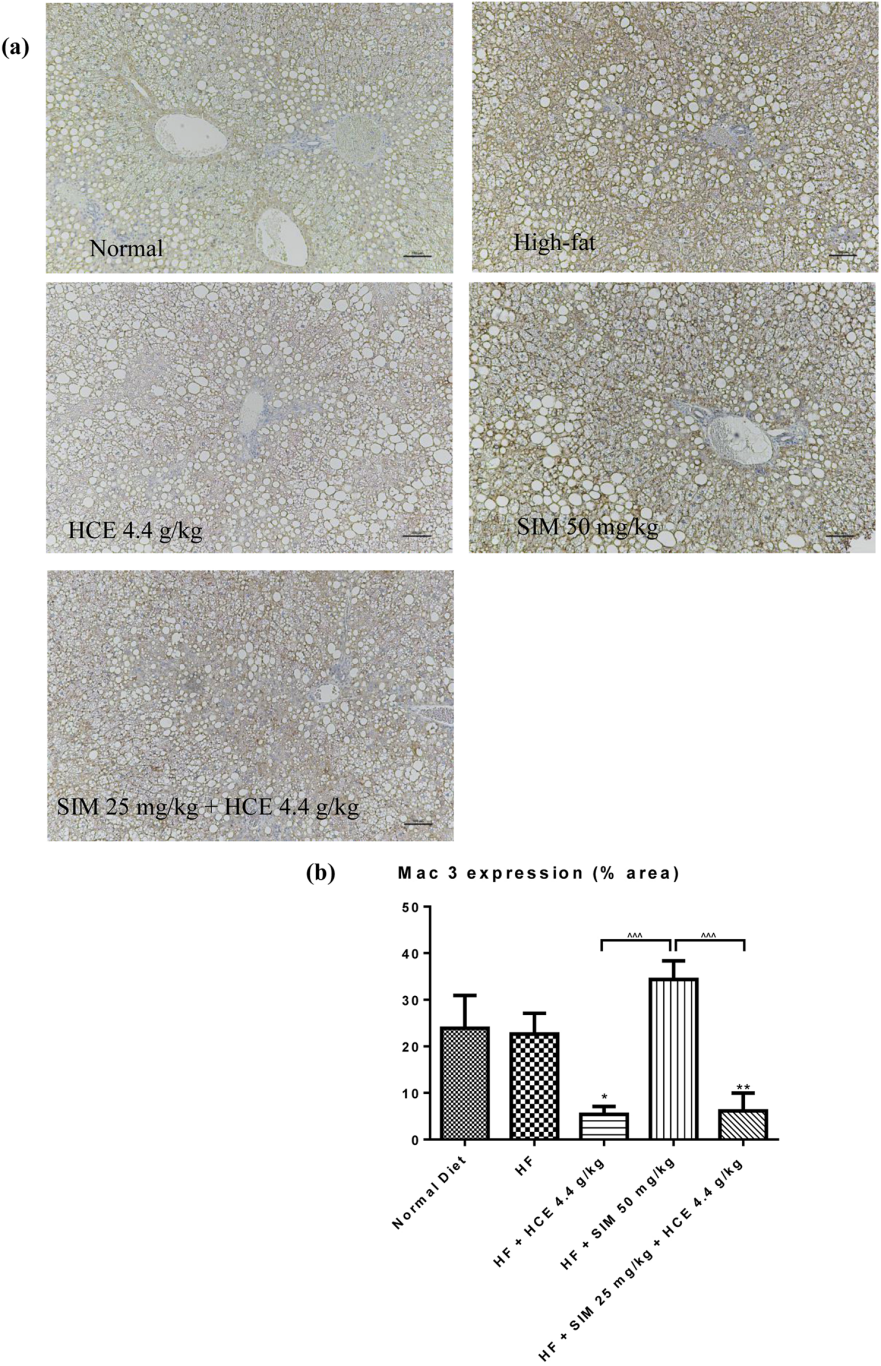
(**a**) Immunohistological staining (x100 magnification) for the presence of macrophage (Mac-3) in liver sections of mice given different treatments and (**b**) percentage area of liver section infiltrated by macrophages, as determined by Mac-3 immunohistological staining in different groups. Values represent means ± SEM (n = 8–10). No significant difference was observed between HF-fed and normal chow-fed group using Student’s *t*-test. Significant difference among HF-fed group vs HF + SIM 50 mg/kg, or HF + HCE 4.4 g/kg, or HF + SIM 25 mg/kg + HCE 4.4 g/kg groups using one-way ANOVA: *p < 0.05, **p < 0.01. Significant difference among HF + SIM 25 mg/kg + HCE 4.4 g/kg, HF + HCE 4.4 g/kg, and HF + SIM 50 mg/kg groups using one-way ANOVA: ^^^^^ *p* < 0.001. The scale bar represents 100 μm.

Immunohistochemical staining was performed to stain for the presence of macrophages (Mac-3) (Fig. 10a). Immunohistochemical analysis of liver sections revealed the presence of Mac-3 in both high-fat-fed and normal chow-fed mice (brown colouration). This inflammation was further increased in simvastatin-treated animals, although such increase did not reach statistical significance. Interestingly, HCE treatment, with or without simvastatin co-treatment significantly reduced inflammation in the high-fat-fed mice. When comparing among all treatment groups, HCE treatment, with or without simvastatin co-treatment also had significantly lower inflammation compared to the simvastatin treatment group (*p* < 0.001 and *p* < 0.001, respectively). These data are also presented as percentage areas in Fig. 10b.

## Discussion

The present study demonstrated that Herba Cistanches water extract (HCE), when co-treated with simvastatin *in viv*o, could a) restore muscle weights including quadriceps and gastrocnemius muscles; b) reduce simvastatin-induced elevated plasma CK; c) improve muscle glutathione levels (antioxidant status); d) restore mitochondrial membrane potential within the muscle; and e) reduce muscle inflammation, therefore further supporting our hypothesis that HCE could exert beneficial effects on simvastatin-induced muscle toxicity. Furthermore, we have also proven for the first time that HCE could exert reduction on: a) liver weight; b) total liver lipid levels; and c) plasma lipid levels and CK in high-fat-fed mice. These data suggested HCE has potential beneficial effects on liver and plasma lipid metabolism in mice with diet-induced obesity. Taken together, our results provided scientific evidence for the first time that HCE not only exhibited potential protective effects on simvastatin-induced toxicity in muscles, but it could also exert beneficial effects on diet-induced non-alcoholic fatty liver and hyperlipidemia when being used alone or in combination with simvastatin at a reduced dose.

It has been previously documented that statin could down-regulate selenoprotein, compromising the antioxidant defense and contributing to its adverse effects^2^. In our *in vivo* muscle toxicity rat model, we observed a significant reduction in GPx activity in muscles of simvastatin-treated rats. However, our muscle gene expression analysis showed that simvastatin-induced significant increase in the expression of genes regulating antioxidants including *GPx1* and *GPx7*, as well as in the expression of other genes regulating ROS metabolism and oxidative stress, possibly due to the fact that muscle cells had undergone oxidative stress as induced by simvastatin, and had therefore in turn upregulated the expressions of GPxs and other oxidative stress responsive genes in response as defense mechanism^23, 27^. On the other hand, HCE alone also significantly increased the gene expression of *GPx1*, suggesting HCE has strong anti-oxidant activity. Furthermore, our results also demonstrated that simvastatin induced a significant reduction in both the muscle mitochondrial MMP and ROS levels. It is plausible that simvastatin induced excessive oxidant stress burden exposure to the muscle cells, causing an increase in ROS levels which reaches a threshold level that triggers the mitochondrial membrane permeability transition, and leading to the collapse of the mitochondrial membrane potential^28–30^. While generated ROS can then be released into cytosol leading to the significant drop of ROS levels within the mitochondion as observed in our study, and causing mitochondrial and cellular injury^28–30^. These observations are in consonance with the findings from previous literatures. Statins were shown to induce reactive oxygen species (ROS) and mitochondrial oxidative stress, as well as acting directly on tissue mitochondria to induce Ca^2+^-dependent membrane permeability transition (MPT)^31, 32^. Interestingly, our HCE could potentially improve the anti-oxidative status, in particularly the GPx activity within the muscle, thereby protecting the cells from oxidative stress-induced collapse in mitochondrial MMP, and ameliorating the side effects as caused by statins. Nonetheless, these hypotheses could be further confirmed by additional mechanistic studies.

The risk of developing statin-induced myopathy is highly associated with the type, dose, lipophilicity, drug interactions of the statin, as well as other patient-related risk factors including age, co-morbidities, gender, genetics, and ethnicity^33^. Increasing studies suggested that statin-induced myopathy is associated with genetic polymorphisms in various drug transporters, autophagy clearance pathways, or enzymes involved in creatine synthesis^34–37^. Mangravite *et al*. recently reported a potential genetic marker which could be responsible for decreased risk of statin-induced myopathy^36^. Glycine amidinotransferase is the rate-limiting enzyme required for creatine biosynthesis. Creatine is primarily synthesized in the liver and kidneys, which then subsequently transported to skeletal muscle to support cellular energy^36^. Nonetheless, not every affected patient is a carrier of such a polymorphism due to human heterogeneity. Additional adequately powered studies of well-characterized statin-induced myopathy cases are warranted to determine their significance^38–40^. This had also led to other putative mechanisms which could possibly be responsible for the statin-induced myopathies, such as disturbed calcium homeostasis, decreased protein prenylation, and increased atrogin-1 expression^34, 41^.

In another recent study, Schirris *et al*. had identified statin lactones, as converted by uridine 5′-diphospho-glucuronosyltransferases (UGTs) from their pharmacologically active acid form in the body upon administration, had played a major role in decreasing the maximal rate of mitochondrial ATP production in C2C12 myoblasts^42^. These statin lactones specifically inhibited the enzymatic activity of CIII of the respiratory chain, and were in general three times more potent inducers of cytotoxicity than their corresponding acid forms^41^. More interestingly, in the muscle biopsy sample of the statin-treated patients, a marked accumulation of the lipophilic statins were observed (acid and lactone) in these patients^41^, it would be worthwhile to determine the levels of statins within the muscle of our animals and understand whether HCE would affect the accumulation of statins in muscles.

Our results demonstrated that HCE could exert beneficial effects on hyperlipidemia that is comparable with simvastatin treatment at the original dose, and the use of HCE together with the reduced dose of simvastatin further reduced the diet-induced hyperlipidemia which appeared to be even more potent than simvastatin treatment alone at its original dose, suggesting a possible additive/synergistic effect between HCE and simvastatin on lipid metabolism. However, this additive effect was only observed on plasma cholesterol and triglyceride levels but not on hepatic cholesterol or triglyceride levels, as evidenced by the lack of significant difference on liver lipid levels between HCE + simvastatin co-treatment group and HCE treatment alone group, or simvastatin treatment alone group. It is possible that HCE exerted additive/synergistic effect with simvastatin only on regulation of plasma lipid metabolism but not on liver lipid metabolism. It would be worthwhile to conduct further herb/drug interaction studies between HCE and simvastatin to determine if HCE could interact with simvastatin to affect the plasma and liver concentrations of simvastatin and/or its metabolites for the control of plasma lipid metabolism.

Previous literature suggested that a closely related species *Cistanche tubulosa* could exert hypocholesterolemic effect on both plasma and liver of mice that were given a high cholesterol diet^43^. Although this study suggested the potential of *Cistanche tubulosa* in reducing plasma and liver TC, it only investigated the effects on cholesterol but not triglyceride. The effect of *Cistanche tubulosa* on other lipid levels has only been investigated more lately in a recent study carried out by Xiong *et al*. which demonstrated that *Cistanche tubulosa* could significantly reduce both plasma TC and TG levels in db/db mice^44^. Nonetheless, this study employed the use of db/db mice which are genetically modified animals which do not directly mimic the real clinical situation whereby hyperlipidemia and non-alcoholic fatty liver disease (NAFLD) are often caused by chronic consumption of the western type diet i.e. high-fat diet. However, there exists no experimental data or detailed study investigating the effects of *Cistanche deserticola* on hyperlipidemia or NAFLD. In order to determine whether Herba Cistanches (*Cistanche deserticola*) treatment to high-fat-fed animals results in an improvement in plasma and liver lipid levels, we carried out the present study in C57BL/6 mice to compare the effects of Herba Cistanches treatment on high-fat diet-induced metabolic syndrome. The potent ability of HCE to reduce both plasma and liver lipid content in obese mice suggests that it might be of therapeutic benefit in humans with diet-induced metabolic syndrome, in particularly for obese individuals with hyperlipidemia and NAFLD. In fact, NAFLD affects 10-20% of the general population and is commonly found in obese or diabetic patients^45^. At present, there is no established treatment for it and current suggested management strategy relies on diet regimen, weight loss and exercise. Dietary supplements/nutraceuticals that might help to delay the development or alleviate this condition are therefore of great importance^46, 47^. Although statins exert the potential to treat hyperlipidemia and possibly reducing liver cholesterol, concerns arise regarding its adverse effects on muscles. Due to the occurrence of these side effects, statins remain underused. The ability of HCE administration to exert potent hypolipidemic effect and therapeutic effect on diet-induced NAFLD has provided great potential of being used as a therapeutic treatment/supplementation targeting diet-induced metabolic syndrome.

In conclusion, this study has provided an insight on the novel approach of using HCE to reduce the muscle toxicity caused by statin, and thus anticipated great benefits in those patients with hyperlipidemia whereby statin prescription is normally not feasible due to muscle toxicity. Furthermore, the hypocholesterolemic effect of HCE provided additional benefits whereby the dosage of statin used could be reduced. Of course, further studies will be needed to investigate the herb-drug interaction and pharmacokinetics between HCE and simvastatin, as well as to determine the safety issue regarding the combination use of HCE and simvastatin in a more chronic term in the clinical setting.
